# DNA methylation in the *APOE* genomic region is associated with cognitive function in African Americans

**DOI:** 10.1186/s12920-018-0363-9

**Published:** 2018-05-08

**Authors:** Jiaxuan Liu, Wei Zhao, Erin B. Ware, Stephen T. Turner, Thomas H. Mosley, Jennifer A. Smith

**Affiliations:** 1 Department of Epidemiology, School of Public Health, University of Michigan, 1415 Washington Heights, 4602 SPH Tower, Ann Arbor, MI 48109-2029 USA; 2 Survey Research Center, Institute for Social Research, University of Michigan, Ann Arbor, MI 48104 USA; 3 Division of Nephrology and Hypertension, Mayo Clinic, Rochester, MN 55905 USA; 4 Department of Neurology, University of Mississippi Medical Center, Jackson, MS 39126 USA

**Keywords:** DNA methylation, Epigenetics, Cognition, Cognitive function, Delayed recall, Memory, Apolipoprotein E

## Abstract

**Background:**

Genetic variations in apolipoprotein E (*APOE*) and proximal genes (*PVRL2*, *TOMM40*, and *APOC1*) are associated with cognitive function and dementia, particularly Alzheimer’s disease. Epigenetic mechanisms such as DNA methylation play a central role in the regulation of gene expression. Recent studies have found evidence that DNA methylation may contribute to the pathogenesis of dementia, but its association with cognitive function in populations without dementia remains unclear.

**Methods:**

We assessed DNA methylation levels of 48 CpG sites in the *APOE* genomic region in peripheral blood leukocytes collected from 289 African Americans (mean age = 67 years) from the Genetic Epidemiology Network of Arteriopathy (GENOA) study. Using linear regression, we examined the relationship between methylation in the *APOE* genomic region and multiple cognitive measures including learning, memory, processing speed, concentration, language and global cognitive function.

**Results:**

We identified eight CpG sites in three genes (*PVRL2*, *TOMM40*, and *APOE)* that showed an inverse association between methylation level and delayed recall, a measure of memory, after adjusting for age and sex (False Discovery Rate q-value < 0.1). All eight CpGs are located in either CpG islands (CGIs) or CGI shelves, and six of them are in promoter regions. Education and *APOE* ε4 carrier status significantly modified the effect of methylation in cg08583001 (*PVRL2*) and cg22024783 (*TOMM40*), respectively. Together, methylation of the eight CpGs explained an additional 8.7% of the variance in delayed recall, after adjustment for age, sex, education, and *APOE* ε4 carrier status. Methylation was not significantly associated with any other cognitive measures.

**Conclusions:**

Our results suggest that methylation levels at multiple CpGs in the *APOE* genomic region are inversely associated with delayed recall during normal cognitive aging, even after accounting for known genetic predictors for cognition. Our findings highlight the important role of epigenetic mechanisms in influencing cognitive performance, and suggest that changes in blood methylation may be an early indicator of individuals at risk for dementia as well as potential targets for intervention in asymptomatic populations.

**Electronic supplementary material:**

The online version of this article (10.1186/s12920-018-0363-9) contains supplementary material, which is available to authorized users.

## Background

Apolipoprotein E (apoE) plays an essential role in lipid metabolism, and variability in this protein influences several lipid-related diseases of older age, including coronary heart disease, stroke, and Alzheimer’s disease (AD) [1]. ApoE also likely influences AD through other biologic mechanisms, including amyloid-β aggregation [2]. The *APOE* ε4 allele has been identified as the most robust genetic risk factor for AD and age-related cognitive impairment in multiple populations including African Americans [3]. In addition to the ε4 allele, other genetic variations in *APOE* and genes in close genomic proximity, such as *TOMM40, PVRL2,* and *APOC1*, are also associated with cognition [4–10] and may contribute to the pathogenesis of age-related cognitive impairment.

Cognition and AD risk may be influenced not only by the DNA sequence but also by epigenetic profiles in the *APOE* region. Epigenetic mechanisms, such as histone modification, DNA methylation, and non-coding RNA can regulate gene expression while the underlying DNA sequence remains the same. The epigenome is influenced both by underlying genetic variants as well as by environmental factors including the social environment, health behaviors, and environmental pollutants [11]. Methylation of CpG dinucleotides, the best understood epigenetic mechanism, is also dynamic over the life course. It is well established that epigenomic patterns of DNA methylation change with age [12]. A recent study in lymphocytes showed that methylation levels at several CpG sites in *APOE* were significantly associated with age, and that these age-related changes in methylation were modified by *APOE* genetic variants [13].

Methylation levels at CpG sites in *APOE* have also been associated with AD or its underlying pathology in the brain. For example, decreased methylation was observed in the CpG island (CGI) nested within the fourth exon of *APOE* in brain tissue from AD cases compared to controls [14]. This differential methylation was only observed in brain regions known to impact AD pathophysiology (frontal lobe and hippocampus), suggesting that *APOE* methylation may play a role in AD pathogenesis. Further, in postmortem brain tissue, a significant positive association was found between methylation at a separate site within *APOE* exon 4 and neuritic amyloid plaque burden, the primary subclinical biomarker of AD pathology [15]. Although these previous studies have explored the relationship between *APOE* methylation and AD, there are still many unanswered questions about the mechanistic actions of *APOE* methylation and its influences on disease. Additionally, the majority of previous epigenetic studies have focused on participants with European or Asian ancestry [12]. To our knowledge, no study has investigated the association between *APOE* methylation and cognitive function in older African Americans without clinically-diagnosed dementia.

In this study, we investigate the association between peripheral blood leukocyte methylation levels in the *APOE* genomic region (*APOE*, *TOMM40*, *PVRL2*, and *APOC1*) and cognitive function in 289 African American participants from the Genetic Epidemiology Network of Arteriopathy (GENOA) study. We also assess whether education and the presence of the *APOE* ε4 allele confound and/or modify the association between methylation and cognitive function. Deeper insight into the epigenetic mechanisms that influence cognitive function may facilitate improved risk assessment, earlier intervention, and more effective treatment for AD and cognitive decline in the era of precision medicine.

## Methods

### Study sample

Study participants are African Americans from the Genetic Epidemiology Network of Arteriopathy (GENOA) study, a community-based study of hypertensive sibships designed to investigate the genetics of hypertension and target organ damage in African Americans from Jackson, Mississippi, and non-Hispanic Whites from Rochester, MN [16, 17]. In the initial phase of GENOA (Phase I: 1996–2001), all members of sibships containing ≥2 individuals with essential hypertension clinically diagnosed before age 60 were invited to participate, including both hypertensive and normotensive siblings. Eighty percent of African Americans (*N* = 1482) from the initial study population returned for the second examination (Phase II: 2001–2005). Hypertension was defined as having systolic blood pressure ≥ 140 mmHg, diastolic blood pressure ≥ 90 mmHg, or previous diagnosis of hypertension and use of antihypertensive medication. Diabetes was defined as fasting blood glucose ≥126 mg/dL or use of antidiabetic medication. Body mass index (BMI, kg/m^2^) was calculated from measured weight and height. A subset of participants also had measures of cerebral white matter hyperintensity (WMH). WMH was assessed by Magnetic Resonance Imaging (MRI), and transformed as ln(WMH + 1) to reduce skewness. In an ancillary study (2001–2006), a total of 1010 African American GENOA participants completed a battery of established cognitive tests that assessed five domains including memory, learning, processing speed, language, and concentration. DNA methylation levels were measured from blood samples collected at Phase I for 422 unrelated participants. Of those with DNA methylation, 289 had at least one cognitive measure and had never been diagnosed with dementia by a physician, and a smaller subset (*N* = 247) also had genotype data available for analysis.

### Cognitive function measures

Six measures of cognitive function were evaluated [18]: (1) the Wechsler Adult Intelligence Scale Revised Digit Symbol Substitution Task (DSST) assessed processing speed, complex visual attention and visuomotor coordination, (2) the Controlled Oral Word Association Test (COWA, Animal Naming) assessed category fluency, (3) the COWA (FAS) assessed letter fluency, (4) the Rey Auditory Verbal Learning Test (RAVLT) immediate memory trial assessed learning ability, (5) the RAVLT delayed memory trial assessed delayed recall, and (6) the Stroop Color and Word Test (SCWT) assessed concentration effectiveness. For additional details, see Smith et al. [19].

Since global cognitive function is an important indicator of health in older age, reflecting an on-the-spot information processing function employed across various cognitive domains [20], we created a measure of global cognitive function using principal component analysis (PCA) in the sample of GENOA African Americans (*N* = 1010) with cognitive data. The global cognitive function factor was defined as the first unrotated principal component from PCA using the covariance structure of the six cognitive measures described above. This factor explained 53% of the total variance of the six cognitive measures, and was well loaded by all six measures (loading values ranged from 0.63 to 0.80).

### DNA methylation measures

DNA methylation was measured using peripheral blood leukocytes from stored blood samples collected during Phase I. The methylation assay was performed at the Mayo Clinic Advanced Genomics Technology Center using the Illumina Infinium HumanMethylation450 BeadChip. The Minfi R package [21] was used to preprocess and normalize the data using the SWAN method [22]. β-values for each CpG site were calculated as β = max(M,0)/[(max(U,0) + max(M,0) + 100], where M represents the fluorescence intensity of the methylated sites and U represents the fluorescence intensity of the unmethylated sites. Individual samples and CpG sites with a high missing rate (> 5%) were excluded. The proportion of each white blood cell type was estimated using Houseman’s method [23]. For each CpG site, the methylation level used for analysis was defined as the β-value multiplied by 100 (described as a percentage of methylation), adjusted for batch effects and white blood cell proportions. β-values greater than four standard deviations from the mean of each CpG site were excluded. Forty-eight CpG sites in the *PVRL2-TOMM40-APOE-APOC1* genomic region were analyzed. We used Illumina annotation [24] to determine whether each CpG site was in a promoter region (within 1.5 kb before the transcription start site of a gene) and/or a CGI, CGI shore (0–2 kb from CGI), or CGI shelf (2-4 kb from CGI).

### Genotype measures

We assessed the presence of the *APOE* ε2 and ε4 alleles in a subset of participants with available genotype data (*N* = 247) to evaluate potential confounding and/or effect modification by *APOE* genotype. DNA was isolated using the PureGene DNA Isolation Kit from Gentra Systems (Minneapolis MN). Genotyping, based on polymerase chain reaction amplification techniques, was conducted at the University of Texas-Health Sciences Center at Houston using the TaqMan assay and ABI Prism® Sequence Detection System (Applied Biosystems, Foster City CA). Participants were classified as ε2 carriers (having at least one copy of ε2 allele) vs. non-carriers, and ε4 carriers vs. non-carriers.

### Statistical analysis

All statistical analyses were performed in SAS version 9.4 (Cary, NC) and RStudio [25]. Characteristics of the full analysis sample (*N* = 289) and the subset sample with genotype data (*N* = 247) were compared using T-tests for continuous variables or χ ^2^ tests for categorical variables.

The primary analysis was conducted in the full analysis sample (*N* = 289). Linear regression was used to test for association between the methylation level of each CpG site and each cognitive measure, adjusting for sex and age at cognitive testing (Model 1). A second set of models also included education (less than high school degree, high school degree/GED, beyond high school degree) (Model 2). We assessed models with and without education because adjustment for education can potentially mask the effect of inherited factors on cognition, if they also influence educational attainment [26]. The coMET package [27] was used to create a regional plot for visualization of the association *P* values. To correct for multiple comparisons, False Discovery Rate (FDR) q-values [28] were calculated for each cognitive measure across the 48 CpG sites, and FDR q < 0.1 was considered significant. For significant associations, models further controlling for hypertension, diabetes, BMI, and current smoking were constructed to test potential confounding by these cognition risk factors. Similarly, in participants with available data for WMH (*N* = 232), we further adjusted the significant associations for hypertension and WMH.

To test the potential confounding effect of the *APOE* ε4 allele on the association between methylation and cognition, a subset analysis was conducted among people with available genotype data (*N* = 247). For Models 1 and 2, ε4 carrier status was added as an adjustment variable. Only CpG sites showing significant association with cognitive measures in the primary analysis (FDR q < 0.1) were further evaluated in the subset analysis. Associations with *P* < 0.05 were considered to remain significant in the subset analysis.

For cognitive measures that showed significant associations with CpG sites after accounting for ε4 (*P* < 0.05 in the subset analysis), we quantified the percent variation in the cognitive measure explained by methylation. We first constructed a full model that included all of the significant CpG sites, plus age, sex, and ε4 carrier status. We then compared this full model to a reduced model that excluded the CpG sites, and calculated the change in *R*^2^. We repeated this analysis including education in both the full and reduced models.

We also examined education and ε4 as potential modifiers of the effect of methylation on cognitive function. For the significant CpG sites in the full sample analysis (FDR q < 0.1), an interaction term between education and methylation was added to Model 2. Similarly, an interaction term for ε4 carrier status and methylation was further added to each of the regression models performed in the subset analysis. Interactions with *P* < 0.05 were considered significant.

### Sensitivity analyses

People with a previous stroke or prodromal dementia may have cognitive changes or methylation alternations that are distinct from those of non-pathological cognitive aging. We used results from the Mini–Mental State Examination (MMSE), a screening tool for dementia [18], to identify participants that may be in the early stages of dementia. In order to assess whether people with previous stroke or preliminary evidence of dementia influenced the estimated association between methylation and cognition, 46 participants with stroke history and/or with an MMSE score of 23 or less [18] were further excluded from the full sample, and the primary analysis was repeated on the significant CpG sites in the remaining 243 participants.

Moreover, since the *APOE* ε2 allele has been previously reported to have a protective effect on cognitive function [29], we also assessed whether the ε2 allele masked the effect of ε4. To evaluate this, we repeated the subset analysis in 186 participants who were non-carriers of the ε2 allele. Associations with *P* < 0.05 were considered to remain significant in the sensitivity analyses.

### Methylation correlations across blood and brain

To investigate whether cognition-related methylation changes identified in this study (FDR q < 0.1) may reflect underlying methylation changes in the brain, we used two publically-available databases that compare blood and brain methylation. The Blood Brain DNA Methylation Comparison Tool (http://epigenetics.essex.ac.uk/bloodbrain/) [30] includes methylation levels in whole blood and four brain regions (prefrontal cortex (PFC), entorhinal cortex (EC), superior temporal gyrus (STG), and cerebellum (CER)) in *N* = 71–75 matched samples from individuals archived in the MRC London Neurodegenerative Disease Brain Bank. This sample includes both neuropathologically unaffected controls and individuals with variable levels of neuropathology. The Blood-Brain Epigenetic Concordance database (BECon; https://redgar598.shinyapps.io/BECon/) [31] includes methylation levels in blood and three cortical regions (Broadmann area 10 (BA10), prefrontal cortex; Broadmann area 7 (BA7), parietal cortex; and Broadmann area 20 (BA20) temporal cortex) from 16 individuals ranging from 15 to 87 years of age from the Douglas-Bell Canada Brain Bank.

## Results

### Sample characteristics

Sample characteristics are shown in Table 1. The range of age at cognitive testing in the full sample was 45 to 85 years (mean = 67), and 71.6% of the participants were female. Participants were relatively evenly distributed across three education levels. Participant characteristics were not significantly different between the full sample and the subset sample (T-test or χ ^2^ test *P* > 0.05 for all characteristics). The *APOE* ε4 allele frequency in the subset sample (0.23) was consistent with previous findings in African Americans [32, 33].

**Table 1:** Characteristics of African Americans from the GENOA study with cognitive and DNA methylation data^a^

		Full analysis sample^b^	Subset sample^c^
		(*N* = 289)	(*N* = 247)
Age (years)		67.2 (7.1)	67.4 (6.8)
Sex (female)		207 (72%)	183 (74%)
Education	Less than high school degree	95 (33%)	83 (34%)
	High school degree / GED	81 (28%)	68 (27%)
	Beyond high school degree	113 (39%)	96 (39%)
Body mass index (BMI, kg/m^2^)		30.9 (6.0)	30.9 (6.1)
Current smoker		34 (12%)	29 (12%)
Diabetes		85 (29%)	70 (28%)
Hypertension		241 (83%)	205 (83%)
White matter hyperintensity (WMH, cm^3^)		12.0 (14.2)	11.9 (13.6)
*APOE* ε4 carrier		N/A	102 (41%)
*APOE* ε2 carrier		N/A	60 (24%)
Processing speed (DSST, symbols)^d^		30.6 (13.2)	30.4 (13.2)
Category fluency (Animal Naming, words)^d^		14.2 (4.4)	14.0 (4.2)
Letter fluency (FAS, words)^d^		28.2 (11.5)	28.1 (11.5)
Learning (RAVLT, words)^d^		38. 5 (9.7)	38.5 (9.5)
Delayed recall (RAVLT, words)^d^		6.1 (3.6)	6.2 (3.6)
Concentration effectiveness (SCWT, items)^d^		20.5 (9.1)	20.4 (8.9)
Global cognitive function		− 0.12 (0.95)	− 0.17 (0.94)

^a^Mean (standard deviation) or n (%) are presented

^b^In the full sample, sample sizes for the cognitive measures range from 243 to 289. A total of 232 participants had data for WMH

^c^Subset sample consists of subjects with available genotype data

^d^Values of cognitive scores are the numbers of symbols, words, or items completed within certain time limits as required by each test. For additional details, see Smith et al. [19]

### Methylation pattern in the *APOE* genomic region

**Fig. 1:**
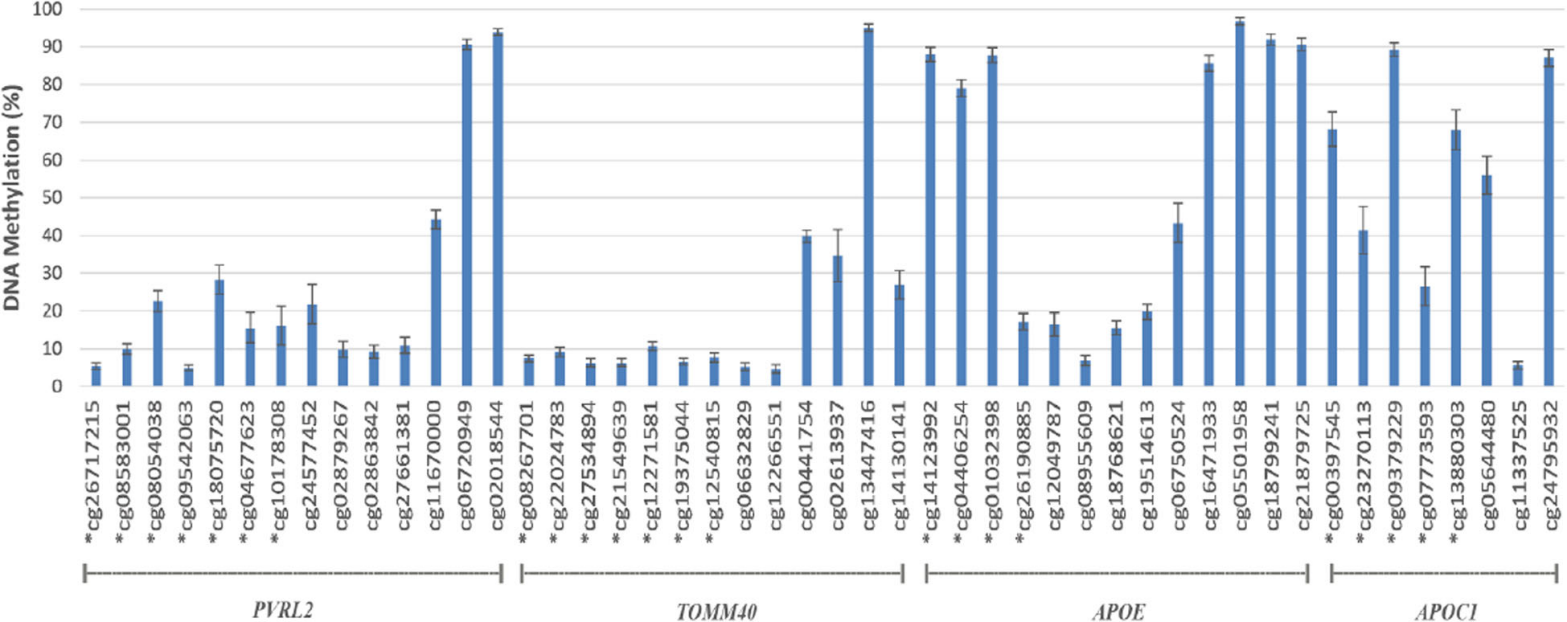
Methylation pattern in the *APOE* region. DNA methylation level (%) is represented by the mean *β* value multiplied by 100 for each of the 48 CpG sites in the *PVRL2-TOMM40-APOE-APOC1* region, adjusted for batch effects and white blood cell proportions (*N* = 289). Error bars represent the standard deviation of methylation level at each CpG site. CpG sites marked with an asterisk (*) are in promoter regions (within 1.5 kb before the transcription start site of the gene)

**Fig. 2:**
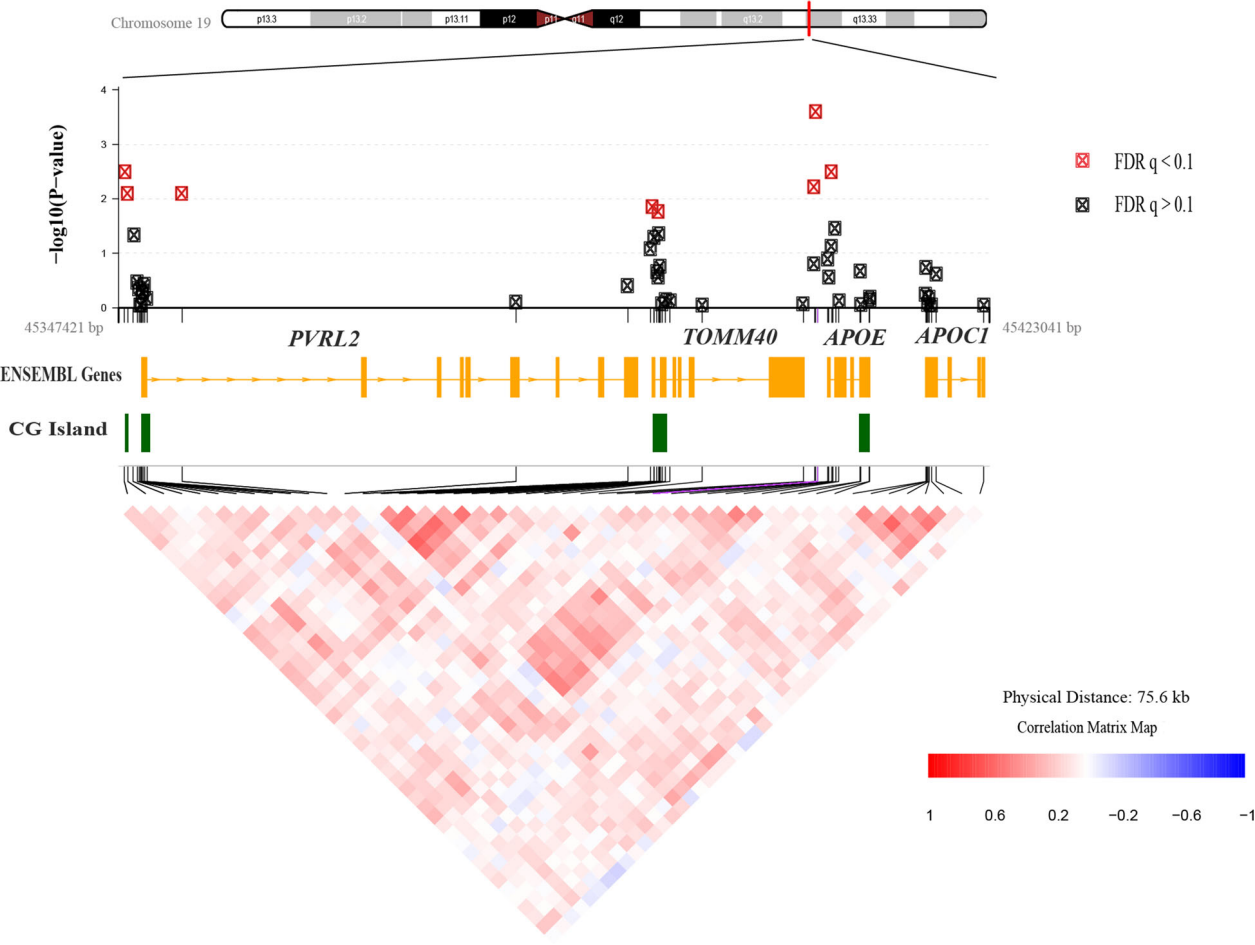
Regional plot of the association between delayed recall and DNA methylation in the *PVRL2-TOMM40-APOE-APOC1* region. The top panel shows -log_10_ (*P* value) for the associations between methylation and delayed recall, adjusting for age and sex, according to chromosomal positions. Significant associations (FDR q < 0.1) are shown in red. The middle panel shows the locations of the genes (Ensembl Genes) and CpG Islands (CG Islands, from UCSC Genome Browser) in this region. The lower panel shows the correlation in DNA methylation levels among the 48 CpG sites in this region

The mean methylation level (%) at each CpG site within the *PVRL2-TOMM40-APOE-APOC1* region is shown in Fig. 1. The variability in methylation levels across individuals was different between CpG sites, with standard deviation ranging from 0.7 to 7%. The mean methylation levels of the 13 CpGs in the *APOE* gene were consistent with previous observations [13]. Specifically, the first three and the last four CpGs had relatively high methylation (> 50%), while the six CpG sites between them had relatively low methylation (< 50%). Among all 48 CpG sites in our study, 15 had relatively high methylation (> 50%), and 23 were located in promoter regions. The CpG sites in the promoter regions of *PVRL2* and *TOMM40* had relatively low methylation (< 50%), while the mean methylation levels of CpG sites in the *APOE* and the *APOC1* promoter regions were more variable. Methylation levels were positively correlated for 82% of the pairs of CpG sites. For all pairs, Pearson correlation coefficients (r) ranged from − 0.15 to 0.59 (Fig. 2).

### Association between methylation and cognitive function

We evaluated the association between DNA methylation of the 48 CpG sites in the *PVRL2-TOMM40-APOE-APOC1* genomic region and cognitive measures in the full analysis sample (*N* = 289). We observed significant inverse associations (FDR q < 0.1) between methylation levels of eight CpG sites and delayed recall (Table 2), including three CpGs in *PVRL2* (cg26717215, cg08583001, and cg11670000), two in *TOMM40* (cg22024783 and cg12271581) and three in *APOE* (cg04406254, cg01032398, and cg18768621). All eight CpGs are located in either CGIs or CGI shelves, and six CpGs are in promoter regions of the three aforementioned genes. The association *P* values for delayed recall on a -log_10_ scale according to the chromosomal positions of CpG sites, as well as the correlations among the tested CpG sites, are shown in a regional plot (Fig. 2). Among the eight CpG sites, methylation levels of the two CpGs in *TOMM40* (cg22024783 and cg12271581) were moderately correlated (Pearson *r* = 0.55), and both were correlated with *APOE* cg18768621 (Pearson *r* = 0.35 for cg22024783, Pearson *r* = 0.41 for cg12271581) and *PVRL2* cg11670000 (Pearson *r* = 0.31 for cg22024783, Pearson *r* = 0.30 for cg12271581). The correlations among the other four CpGs were weak (*r* < 0.3).

**Table 2:** Association between DNA methylation and delayed recall for significant CpGs in the full analysis sample (*N* = 282)^a^

	*PVRL2*						*TOMM40*				*APOE*					
	cg26717215		cg08583001		cg11670000		cg22024783		cg12271581		cg04406254		cg01032398		cg18768621	
Region feature(s)^b^	Promoter, CGI		Promoter, CGI		CGI shelf		Promoter, CGI		Promoter, CGI		Promoter, CGI shelf		Promoter, CGI shelf		CGI shelf	
	*β (q)*	∆*R*^*2*^	*β (q)*	*∆R* ^*2*^	*β (q)*	∆*R*^*2*^	*β (q)*	∆*R*^*2*^	*β (q)*	∆*R*^*2*^	*β (q)*	∆*R*^*2*^	*β (q)*	∆*R*^*2*^	*β (q)*	∆*R*^*2*^
Model 1^c^	−0.66 (0.048)	0.03	− 0.37 (0.086)	0.02	− 0.20 (0.086)	0.02	−0.40 (0.086)	0.02	−0.43 (0.086)	0.02	−0.23 (0.086)	0.02	−0.38 (0.012)	0.04	−0.32 (0.048)	0.03
Model 2^d^	−0.62 (0.058)	0.03	−0.42 (0.058)	0.03	−0.19 (0.120)	0.02	−0.41 (0.086)	0.02	−0.39 (0.121)	0.02	−0.23 (0.086)	0.02	−0.35 (0.026)	0.03	−0.29 (0.058)	0.02

^a^In the full sample, 282 participants had non-missing delayed recall measures. Only CpG sites that had a significant association with delayed recall (FDR q < 0.1) after adjustment for age and sex (Model 1) are shown. *β* (q) represents the estimated change in delayed recall score for a 1% increase in methylation level of the CpG site, after adjustment for model covariates, and its associated q-value. ∆*R*^*2*^ is the additional percent variation in delayed recall explained by the CpG site in the model, compared to the reduced model with only covariates

^b^Region feature(s) are from Illumina annotation. “Promoter” = within 1.5 kb before the transcription start site of a gene; “CGI” = within a CpG island; “CGI shelf” = 2-4 kb from CGI

^c^Model 1: Delayed recall = CpG methylation + age + sex

^d^Model 2: Delayed recall = CpG methylation + age + sex + education

After further adjustment for education, the estimated effect of methylation was mildly attenuated for six of the eight CpG sites, except for *PVRL2* cg08583001 and *TOMM40* cg22024783, where effect estimates increased; six of the eight CpG sites remained significantly associated with delayed recall (FDR q < 0.1), with the exception of *PVRL2* cg11670000 (FDR q = 0.120) and *TOMM40* cg12271581 (FDR q = 0.121) (Table 2). All associations remained significant in models further controlling for hypertension and WMH, as well as in models further controlling for hypertension, diabetes, BMI and current smoking (*P* < 0.05, data not shown). The methylation levels of the eight CpG sites were also inversely associated with learning and global cognitive function, adjusted for age, sex, and education; however, the associations were not significant after correction for multiple testing (data not shown). No associations between CpG methylation and any of the other cognitive measures were significant after multiple testing correction (data not shown).

When we repeated the analysis for the eight significant CpG sites in the subset sample with available genotypes (*N* = 242), all eight CpG sites remained significant at *P* < 0.05, except for *APOE* cg04406254 with a marginal significance (*P* = 0.05), and effect estimates for some CpGs were slightly attenuated after adjustment for *APOE* ε4 carrier status (Additional file 1: Table S1). Together the eight CpG sites explained an additional 9.1% of the variance in delayed recall after accounting for age, sex and *APOE* ε4, and 8.7% when further adjusted for education.

### Education and *APOE* ε4 carrier status as effect modifiers

**Fig. 3:**
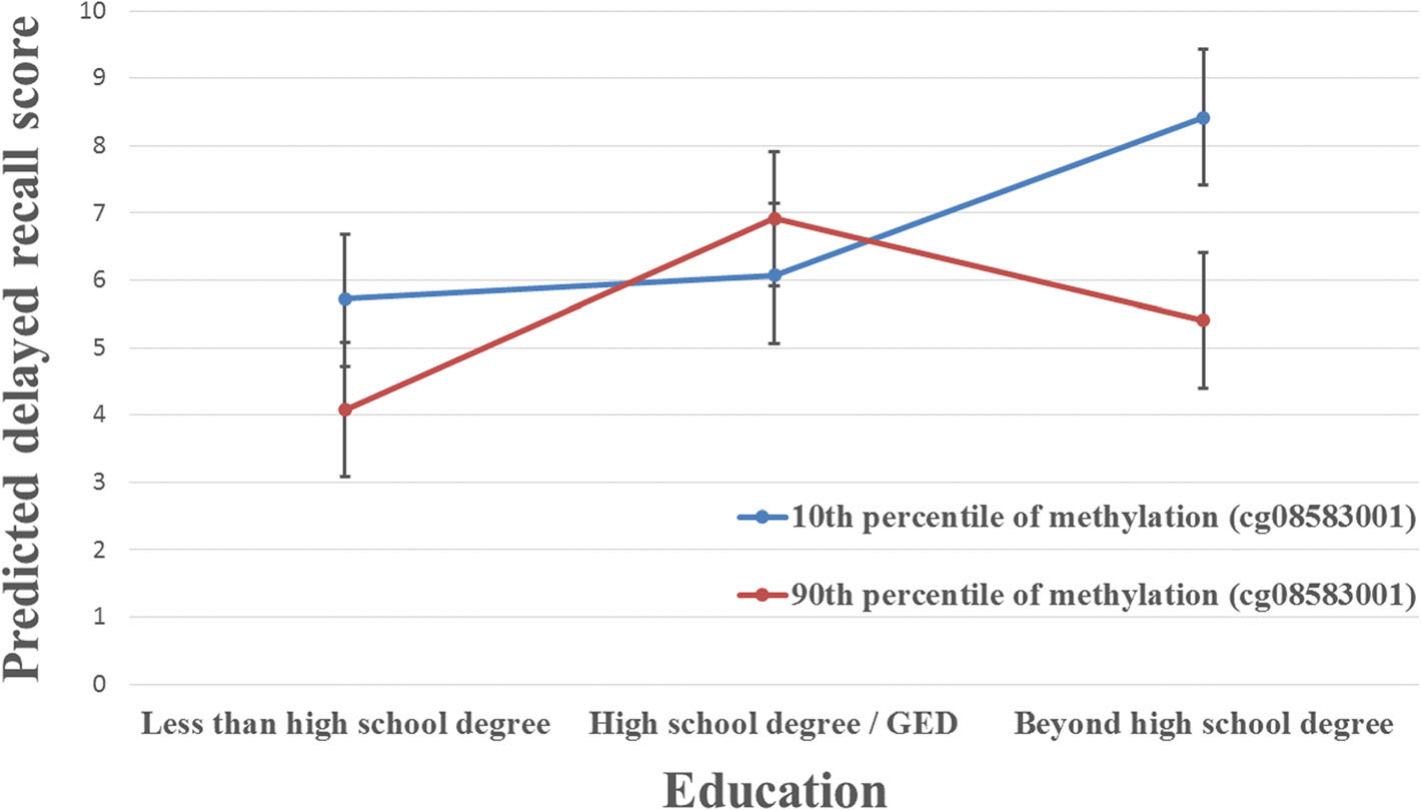
Interaction between *PVRL2* cg08583001 and education on delayed recall. Predicted delayed recall scores were calculated based on the average age and sex distribution in the full analysis sample (*N* = 282), and compared between the 10th and the 90th percentiles of methylation at *PVRL2* cg08583001 by education level. Error bars denote the 95% confidence interval for each predicted delayed recall score

A significant interaction was observed between *PVRL2* cg08583001 and education on delayed recall (*P* for interaction = 0.0046), adjusted for age and sex. Figure 3 shows predicted delayed recall scores calculated for the average age and sex distribution in the full analysis sample (*N* = 282), and compared between the 10th and 90th percentiles of methylation at *PVRL2* cg08583001, by education level. The negative effect of increased *PVRL2* cg08583001 methylation on delayed recall was only significant in those with education beyond a high school degree. However, for participants with high school degree/GED or less, there was no significant association between methylation and delayed recall, as demonstrated by the overlap between the 95% confidence intervals of predicted delayed recall scores for the 10th and the 90th percentiles of methylation. This interaction remained significant (*P* for interaction < 0.05) after further controlling for hypertension and WMH, as well as after controlling for hypertension, diabetes, BMI and current smoking (data not shown).

**Fig. 4:**
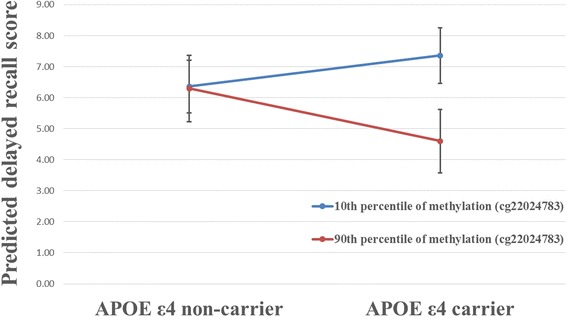
Interaction between *TOMM40* cg22024783 and *APOE* ε4 carrier status on delayed recall. Predicted delayed recall scores were calculated based on the average age and sex distribution in the subset sample (*N* = 242), and compared between the 10th and the 90th percentiles of methylation at *TOMM40* cg22024783 by *APOE* ε4 carrier status. Error bars denote the 95% confidence interval for each predicted delayed recall score

In the subset analysis for individuals with genotype information (*N* = 242), a significant interaction was also observed between *TOMM40* cg22024783 and *APOE* ε4 carrier status (*P* for interaction = 0.013) on delayed recall, adjusted for age and sex. Figure 4 shows predicted delayed recall scores calculated for the average age and sex distribution in the subset sample, and compared between the 10th and 90th percentiles of methylation at *PVRL2 TOMM40* cg22024783, by *APOE* ε4 carrier status. The negative effect of increased *TOMM40* cg22024783 methylation on delayed recall was only observed for *APOE* ε4 carriers, but not for non-carriers. Adjusting for education did not substantively change the results shown in Fig. 4. This interaction remained significant (*P* for interaction < 0.05) after further controlling for hypertension and WMH, and after further controlling for hypertension, diabetes, BMI and current smoking (data not shown).

### Sensitivity analyses

All eight CpG sites significantly associated in the primary analysis remained at least nominally significantly associated with delayed recall (*P* < 0.05) after excluding people with stroke history or MMSE ≤23 (Additional file 2: Table S2). In the sensitivity analysis that excluded *APOE* ε2 allele carriers, results were largely consistent with the analysis that included ε2 allele carriers, although a few CpGs lost significance due to the reduced sample size (Additional file 3: Table S3). Given the similarity of results before and after excluding those with previous stroke, preliminary evidence of dementia, and/or the ε2 allele, it is likely that these factors did not substantively influence the analysis findings.

### Methylation correlations across blood and brain

Correlation between methylation levels in the blood and brain tissue for the eight CpGs associated with delayed recall (FDR q < 0.1) based on data from publically-available databases is shown in Additional file 4: Figure S1 and Additional file 5: Figure S2. Results from both databases show that overall methylation levels are relatively consistent across the tissue types (Additional file 4: Figure S1 and Additional file 5: Figure S2 (panel 1)). That is, CpG sites that are highly methylated (> 50% methylation) in blood are also highly methylated in multiple brain regions. Inter-individual variations tended to be positively correlated between blood and brain for most of the significant CpG sites evaluated. Correlations between blood and the four evaluated regions using the Blood Brain DNA Methylation Comparison Tool ranged from − 0.275 to 0.312, but 23 of the 32 tested correlations (72%) were positive (Additional file 4: Figure S1). Using BECon, correlations between blood and the three evaluated regions ranged from − 0.56 to 0.45, but 15 out of the 24 tested correlations (63%) were positive (Additional file 5: Figure S2 (panel 2)).

## Discussion

While previous studies have suggested that epigenetic modifications may contribute to AD [14, 15], very little is known about the association between DNA methylation and age-related cognitive impairment in those without dementia. To our knowledge this study is the first assessment of the association between DNA methylation and cognitive function in a non-AD population. Studies such as this may enhance understanding of the epigenetic profile for cognitive aging as well as contribute to earlier and novel treatment or intervention strategies for aging-related impairments in cognitive function.

We assessed DNA methylation in the *PVRL2-TOMM40-APOE-APOC1* genomic region, which comprises a moderate to strong linkage disequilibrium (LD) block. Many SNPs in this region, in all four of the genes, have been found to be associated with AD or cognitive impairment [4–10]. It is still unclear whether the majority of the detected associations between the three neighboring genes and cognitive impairment are independent of *APOE* genetic variation or driven at least partially by LD with *APOE* [5, 6]. Each of the three neighboring genes encodes proteins with biological functions that could plausibly influence cognitive function or AD pathogenesis [9]. Specifically, *PVRL2* encodes a cell surface protein involved in the entry activity of herpes simplex virus type 1 (HSV-1) [34, 35]. HSV-1 DNA has been detected in the brains of both healthy elderly and AD cases [36, 37], and has been shown to be a risk factor for AD in ε4 carriers [38]. *TOMM40* encodes a pore subunit of the mitochondrial outer membrane protein translocator, and mitochondrial dysfunction plays an important role in the early pathology of AD [39, 40]. *APOC1* encodes an apolipoprotein involved in a variety of biological processes including cholesterol metabolism and neuronal apoptosis [41].

Previous functional genomic analysis indicated that genetic variants in this region may have regulatory effects, and gene expression patterns within the region may be modulated by a complex transcriptional regulatory structure [42]. The four genes in this region may be mutually influenced by each other through *cis*-elements, supporting the claim that it may be inaccurate to attribute all of this region’s genetic influences on AD and cognition to *APOE* alone [8]. We identified eight CpG sites in three genes within this genomic region (*PVRL2*, *TOMM40,* and *APOE*) that had methylation levels significantly associated with delayed recall. Among the eight CpG sites, methylation levels of the two CpGs in *TOMM40* (cg22024783 and cg12271581) were moderately correlated, and both were correlated with *APOE* cg18768621 and *PVRL2* cg11670000. These moderate correlations provide evidence for the underlying co-regulation structure in this region, which may be due to underlying patterns of LD as well as shared response to environmental stimuli. The remaining four CpG sites were only weakly correlated (*r* < 0.3), suggesting that there may also be a role for more site-specific effects on cognition and/or gene expression.

While fewer than half of the 48 CpGs we explored are located in promoter regions, the majority of the significant CpGs (six out of eight) are promoter-nested sites (Table 2), suggesting that the promoter regions of *PVRL2*, *TOMM40,* and *APOE* may have important epigenetic regulatory functions. Also, as shown by the ENCODE regulation track on UCSC Genome Browser (http://genome.ucsc.edu/), signals for common histone marks including H3K4Me3, H3K4Me1, and H3K27Ac have been detected in and around the promoters of these genes, indicating hotspots for histone modification and chromatin remodeling within these regions. Moreover, all of the significant CpG sites are located in either CGIs or CGI shelves (Table 2). However, no significant results were found for *APOC1*, a gene without a CGI (Fig. 2). Enriched with high CpG content, CGIs have important regulatory function, influencing transcription activity and local chromatin configuration [43]. In addition, previous studies have indicated that CGI flanking regions (both CGI shores and shelves) may also be involved in gene regulation and disease pathophysiology [44, 45]. While most of the CGIs in the human genome are nested in promoter regions, the *APOE* gene is among the gene group that does not possess a classical promoter CGI, but a 3’-end CGI in the exon region [46]. A previous study showed two regions within this 3’-CGI in which AD cases had lower methylation levels than controls, and the difference was only significant in brain regions profoundly impacted by AD pathophysiology (frontal lobe and hippocampus) [14]. In our study, we found three significant CpGs in the shelf of the *APOE* 3’-CGI, but none in the CGI itself. This is possibly because only three of the 90 CpGs in the *APOE* 3’-CGI are present on the Illumina 450K chip, and the inter-individual variation within the 3’-CGI has been shown to be low [46]; thus the 48 CpGs sites analyzed in this study may not necessarily represent the other unmeasured sites.

Significant associations (FDR q < 0.1) between methylation and cognitive measures were only observed for delayed recall, but not for any of the other cognitive measures. Nevertheless, the methylation levels of the eight CpG sites were also consistently inversely associated with learning and global cognitive function, and most of the *P* values were significant at a nominal level (*P* < 0.05), although they became insignificant after correction for multiple testing. It has been suggested that DNA methylation has a critical role for maintaining normal hippocampal function, and may contribute to age-related changes in synaptic plasticity and memory function [47, 48]. Moreover, as reported in previous population studies on cognitive impairment or dementia, while the *APOE* ε4 allele has been associated with various cognitive domains, the strongest association was observed for memory [29]. Our similar findings on methylation in the *APOE* region are in accord with previous studies, indicating that the detrimental effects of *APOE* and/or other neighboring genes may be more pronounced on memory than other cognitive functions.

The effects of methylation in the *APOE* genomic region on cognitive function may be mediated by alterations in gene expression. Increased DNA methylation, especially in promoter regions, often results in repressed gene expression. DNA methylation may repress gene expression by either blocking access of transcriptional factors or recruiting methyl-CpG-binding proteins, which can induce further histone modifications and altered chromatin status [49]. In line with this, data from Encyclopedia of DNA Elements (ENCODE) consortium suggested a negative correlation between methylation in the *APOE* gene (including the three CpGs showing significance in our study) and gene expression in multiple cell lines [13]. Previous studies have also shown an association between down-regulated gene expression in this region and decreased cognitive function. For example, lower plasma level apoE has been reported to be associated with lower cognitive function [50] and higher risk of dementia [51]. In brain tissue from AD cases, *APOE* mRNA level was inversely associated with pathology load, and a separate sample of probable AD cases had a 60% decrease in *APOE* mRNA level in lymphocytes compared to controls [52]. A trend of downregulation of *TOMM40* expression was also observed in AD brains as well as blood samples compared with controls [53]. In line with these findings, our study also showed that increased methylation in the promoters of genes in this region is associated with decreased memory function. However, we did not have the ability to assess the relationship between methylation and gene expression levels in this cohort. Future studies on methylation-gene expression and gene expression-cognition associations, in populations both with and without AD, will help validate the functional effects of methylation in the *APOE* region.

For the majority of the detected associations between methylation and delayed recall, adjustment for education tended to attenuate the estimated effect of methylation, but the changes were small (Table 2). However, we found that education level modified the effect of *PVRL2* cg08583001 methylation on delayed recall, such that the deleterious effect of methylation was only present for individuals with education beyond high school. Additionally, we found a significant interaction between *TOMM40* cg22024783 and being a carrier of the *APOE* ε4 allele. Overall, these findings suggest that the methylation effect is modified by both genetic factors as well as environmental factors. The underlying biological pathways for these interactions will be an important area of future research.

Our study showed that eight CpG sites in the *PVRL2-TOMM40-APOE-APOC1* genomic region explained nearly 10 % of the variance in the delayed recall measure, after accounting for age, sex, education, and *APOE* ε4. Similarly, a previous epigenome-wide association study (EWAS) reported that 71 significant CpGs together have a higher explanatory power (28.7%) for AD pathologic burden than known genetic risk variants (13.9%) [54]. These findings highlight the important role of epigenetic mechanisms in phenotypic diversity and disease pathophysiology. Future work expanding to other gene regions known to be associated with cognitive impairment, as well as the regions discovered in EWAS studies for AD [3] will improve our understanding of the epigenetic structure of cognitive function.

Our study has several strengths. To our knowledge, this is the first study to assess the association between *APOE* methylation and cognitive function in a population-based cohort of older adults without diagnosed dementia. In addition, our research was conducted in African Americans, an under-studied population with high prevalence of dementia and high allele frequency of *APOE* ε4 [33]. Furthermore, with rich cognitive measures, we were able to assess methylation associations with multiple cognitive domains as well as global cognitive function. Last, while most studies have only focused on the *APOE* gene, we also evaluated DNA methylation in the entire LD block surrounding *APOE*, providing new insights into the epigenetic profiles of this genomic region.

This study also has limitations. First, although the GENOA cohort has substantially more African Americans than previous epigenetic studies of the *APOE* region and cognition, the relatively small sample size of participants with both methylation and cognitive measures limited our ability to examine additional regions across the genome. Second, our findings in blood leukocytes provide only limited information on the potential underlying pathological processes in the brain. Recently, it was shown that blood and brain methylation patterns may be quite distinct, and that inferences may not hold across these tissues [55, 56]. Nevertheless, for the eight significant CpGs detected in our study, consistent methylation patterns were found between blood and brain, and inter-individual variations are positively correlated between blood and brain tissue for the majority of the significant CpGs. While not without limitations, methylation studies of complex neurobiological phenotypes in peripheral tissues are essential for identifying biomarkers that precede dementia and serve as a critical compliment to studies of brain tissue, which can only be obtained after death [30]. Third, our study has limited implications for causality because we did not evaluate longitudinal measures of methylation and cognitive function. Since both methylation and cognition are dynamic over time, further longitudinal studies are necessary to investigate how cognitive function changes with alterations in methylation. Finally, multi-level “omic” approaches that integrate dense genetic, epigenetic, and transcriptomic data from this region will improve our understanding of its genetic influence on cognition and dementia.

## Conclusion

In summary, we identified eight CpG sites in the *PVRL2-TOMM40-APOE-APOC1* genomic region that showed inverse association between methylation and delayed recall, after accounting for age and sex. Education and *APOE* ε4 modified the effect of methylation at certain CpG sites. Our findings highlight the important role of epigenetic mechanisms in influencing cognitive performance, and suggest that changes in methylation may be fruitful for early identification of individuals at risk for dementia as well as potential targets for intervention in asymptomatic populations.

## Additional files

Additional file 1: **Table S1.** Association between DNA methylation and delayed recall in the subset sample (*N* = 242). A summary of results for the analysis in subjects with available genotype data, including association coefficients, significance levels, and additional percent variation in delayed recall explained by methylation. (DOC 190 kb)

Additional file 2: **Table S2.** Sensitivity analysis for the association between DNA methylation and delayed recall in subjects without stroke or dementia (*N* = 239). A summary of results for the sensitivity analysis in subjects without stroke history and/or MMSE≤23, including association coefficients and significance levels. (DOC 294 kb)

Additional file 3: **Table S3.** Sensitivity analysis for the association between methylation and delayed recall in *APOE* ε2 non-carriers (*N* = 183). A summary of results for the sensitivity analysis in *APOE* ε2 non-carriers, including association coefficients and significance levels. (DOC 185 kb)

Additional file 4: **Figure S1.** Correlation of blood and brain methylation for the eight CpG sites significantly associated with delayed recall in GENOA: Results from the Blood Brain DNA Methylation Comparison Tool. Boxplots of methylation level by tissue type, and scatterplots demonstrating the relationship between methylation in whole blood and four brain regions (prefrontal cortex (PFC), entorhinal cortex (EC), superior temporal gyrus (STG), and cerebellum (CER)) in *N* = 71–75 matched samples from individuals archived in the MRC London Neurodegenerative Disease Brain Bank. Samples from both neuropathologically unaffected controls and individuals with variable levels of neuropathology were included. Plots were generated from the Blood Brain DNA Methylation Comparison Tool (http://epigenetics.essex.ac.uk/bloodbrain/). Only CpG sites that showed a significant association with delayed recall (FDR q < 0.1) in the GENOA sample were investigated. (PDF 595 kb)

Additional file 5: **Figure S2.** Correlation of blood and brain methylation for the eight CpG sites significantly associated with delayed recall in GENOA: Results from Blood-Brain Epigenetic Concordance (BECon). Comethylation plots showing the methylation levels in blood and three cortical regions (Broadmann area 10 (BA10), prefrontal cortex; Broadmann area 7 (BA7), parietal cortex; and Broadmann area 20 (BA20) temporal cortex) in 16 individuals ranging from 15 to 87 years of age from the Douglas-Bell Canada Brain Bank (panel 1), as well as a summary of blood-brain correlations (Spearman correlation value), variability of the CpGs (range of beta values between 10th and 90th percentile in the sample), and the effect of cell composition (change in beta value before and after adjustment for cell composition) (panel 2). Plots were generated from the Blood-Brain Epigenetic Concordance database (https://redgar598.shinyapps.io/BECon/). Only CpG sites that showed a significant association with delayed recall (FDR q < 0.1) in the GENOA sample were investigated. (PDF 21 kb)

## Abbreviations

AD: Alzheimer’s disease
*APOE*: Apolipoprotein E gene
apoE: Apolipoprotein E protein
BMI: Body mass index
CGI: CpG island
COWA: Controlled Oral Word Association Test
DSST: Digit Symbol Substitution Task
EWAS: Epigenome-wide association study
FDR: False discovery rate
GENOA: Genetic Epidemiology Network of Arteriopathy
HSV-1: Herpes simplex virus type 1
LD: Linkage disequilibrium
MMSE: Mini–Mental State Examination
PCA: Principal component analysis
RAVLT: Rey Auditory Verbal Learning Test
SCWT: Stroop Color and Word Test
SWAN: Subset-quantile within array normalization
