# Supplementary material for: DNA methylation in the APOE genomic region is associated with cognitive function in African Americans
Source: BMC Med Genomics. 2018 May 8;11:43. doi: 10.1186/s12920-018-0363-9 (PMC5941603; doi:10.1186/s12920-018-0363-9)
Supplement: Supplementary file 1 — Table S1. Association between DNA methylation and delayed recall in the subset sample (N = 242). A summary of results for the analysis in subjects with available genotype data, including association coefficients, significance levels, and additional percent variation in delayed recall explained by methylation. (DOC 190 kb) [file 12920_2018_363_MOESM1_ESM.doc]

**Table S1.** Association between DNA methylation and delayed recall in the subset sample (N=242)^a^

|  | ***PVRL2*** | | | | | | | |  | ***TOMM40*** | | | |  | ***APOE*** | | | | | | |
| --- | --- | --- | --- | --- | --- | --- | --- | --- | --- | --- | --- | --- | --- | --- | --- | --- | --- | --- | --- | --- | --- |
|  | **cg26717215** | | **cg08583001** | | | **cg11670000** | | |  | **cg22024783** | | **cg12271581** | |  | **cg04406254** | | **cg01032398** | | | **cg18768621** | |
|  | 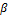 | *∆R^2^* | | 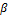 | *∆R^2^* | | 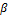 | *∆R^2^* |  | 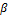 | *∆R^2^* | 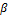 | *∆R^2^* |  | 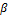 | *∆R^2^* | 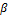 | *∆R^2^* | | 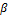 | *∆R^2^* |
| **Model 1^b^** | -0.61** | 0.02 | | -0.42** | 0.03 | | -0.21* | 0.02 |  | -0.39* | 0.02 | -0.52** | 0.03 |  | -0.20* | 0.02 | -0.35** | 0.04 | -0.35** | | 0.03 |
| **Model 1 + ε4**^d^ | -0.60* | 0.02 | | -0.42** | 0.03 | | -0.20* | 0.02 |  | -0.38* | 0.02 | -0.50** | 0.02 |  | -0.19* | 0.01 | -0.34** | 0.03 | -0.34** | | 0.03 |
| **Model 2^c^** | -0.57* | 0.02 | | -0.46** | 0.03 | | -0.20* | 0.02 |  | -0.41* | 0.02 | -0.48** | 0.02 |  | -0.19* | 0.01 | -0.33** | 0.03 | -0.33** | | 0.03 |
| **Model 2 + ε4**^d^ | -0.56* | 0.02 | | -0.46** | 0.03 | | -0.19* | 0.02 |  | -0.41* | 0.02 | -0.47* | 0.02 |  | -0.19 | 0.01 | -0.32** | 0.03 | -0.32** | | 0.03 |
| ^a^ In the subset sample (N=247), 242 participants had non-missing delayed recall measures. Only CpG sites that had a significant association with delayed recall (FDR q<0.1) after adjustment for age and sex (Model 1) in the full sample (N=282) are shown. 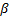 represents the estimated change in delayed recall score for a 1% increase in methylation level of the CpG site, after adjustment for model covariates. *∆R^2^* is the additional percent variation in delayed recall explained by the CpG site in the model, compared to the reduced model with only covariates.  ^b^ Model 1: Delayed recall = CpG methylation + age + sex.  ^c^ Model 2: Delayed recall = CpG methylation + age + sex + education.  ^d^ “ε4”: *APOE* ε4 carrier status.  **P*<0.05, ***P*<0.01 | | | | | | | | | | | | | | | | | | | | | |
