# Supplementary material for: DNA methylation in the APOE genomic region is associated with cognitive function in African Americans
Source: BMC Med Genomics. 2018 May 8;11:43. doi: 10.1186/s12920-018-0363-9 (PMC5941603; doi:10.1186/s12920-018-0363-9)
Supplement: Supplementary file 2 — Table S2. Sensitivity analysis for the association between DNA methylation and delayed recall in subjects without stroke or dementia (N = 239). A summary of results for the sensitivity analysis in subjects without stroke history and/or MMSE≤23, including association coefficients and significance levels. (DOC 294 kb) [file 12920_2018_363_MOESM2_ESM.doc]

**Table S2.** Sensitivity analysis for the association between DNA methylation and delayed recall in subjects without stroke or dementia (N=239)^a^

|  | ***PVRL2*** | | | | | |  | ***TOMM40*** | | | |  | ***APOE*** | | | | | |
| --- | --- | --- | --- | --- | --- | --- | --- | --- | --- | --- | --- | --- | --- | --- | --- | --- | --- | --- |
|  | **cg26717215** | | **cg08583001** | | **cg11670000** | |  | **cg22024783** | | **cg12271581** | |  | **cg04406254** | | **cg01032398** | | **cg18768621** | |
|  | 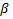 | 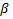 | | 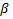 | |  | | | 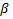 | | 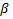 |  | | 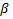 | | 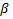 | | 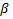 |
| **Model 1^b^** | -0.66** | -0.37* | | -0.20* | |  | | | -0.36* | | -0.49** |  | | -0.21* | | -0.39*** | | -0.24* |
| **Model 2^c^** | -0.66** | -0.43** | | -0.20* | |  | | | -0.39* | | -0.48** |  | | -0.20* | | -0.37*** | | -0.24* |
| ^a^ Among the 243 participants eligible for this sensitivity analysis, 239 had non-missing delayed recall measures. Only CpG sites that had a significant association with delayed recall (FDR q<0.1) after adjustment for age and sex (Model 1) in the full sample (N=282) are shown. 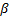 *(P)* represents the estimated change in delayed recall score for a 1% increase in methylation level of the CpG site, after adjustment for model covariates.  ^b^ Model 1: Delayed recall = CpG methylation + age + sex.  ^c^ Model 2: Delayed recall = CpG methylation + age + sex + education.  **P*<0.05, ***P*<0.01, ****P*<0.001 | | | | | | | | | | | | | | | | | | |
