# Supplementary material for: DNA methylation in the APOE genomic region is associated with cognitive function in African Americans
Source: BMC Med Genomics. 2018 May 8;11:43. doi: 10.1186/s12920-018-0363-9 (PMC5941603; doi:10.1186/s12920-018-0363-9)
Supplement: Supplementary file 3 — Table S3. Sensitivity analysis for the association between methylation and delayed recall in APOE ε2 non-carriers (N = 183). A summary of results for the sensitivity analysis in APOE ε2 non-carriers, including association coefficients and significance levels. (DOC 185 kb) [file 12920_2018_363_MOESM3_ESM.doc]

|  | ***PVRL2*** | | | | | |  | | ***TOMM40*** | | | |  | ***APOE*** | | | | | |
| --- | --- | --- | --- | --- | --- | --- | --- | --- | --- | --- | --- | --- | --- | --- | --- | --- | --- | --- | --- |
|  | **cg26717215** | **cg08583001** | | **cg11670000** | | |  | | **cg22024783** | | **cg12271581** | |  | **cg04406254** | | **cg01032398** | | **cg18768621** | |
|  | 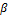 | | 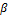 | | 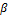 |  | | 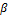 | | 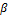 | |  | | | 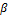 | | 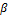 | | 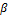 |
| **Model 1^b^** | -0.61** | | -0.42** | | -0.21* |  | | -0.39* | | -0.52** | |  | | | -0.20* | | -0.35** | | -0.35** |
| **Model 1 + ε4**^d^ | -0.70* | | -0.44** | | -0.19 |  | | -0.32 | | -0.52* | |  | | | -0.19 | | -0.35** | | -0.34* |
| **Model 2**^c^ | -0.57* | | -0.46** | | -0.20* |  | | -0.41* | | -0.48** | |  | | | -0.19* | | -0.33** | | -0.33** |
| **Model 2 + ε4**^d^ | -0.65* | | -0.41* | | -0.18 |  | | -0.36 | | -0.47* | |  | | | -0.19 | | -0.33** | | -0.31* |
| ^a^ Among the 186 participants eligible for this sensitivity analysis, 183 had non-missing delayed recall measures. Only CpG sites that had a significant association with delayed recall (FDR q<0.1) after adjustment for age and sex (Model 1) in the full sample (N=289) are shown. 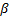 represents the estimated change in delayed recall score for a 1% increase in methylation level of the CpG site, after adjustment for model covariates.  ^b^ Model 1: Delayed recall = CpG methylation + age + sex.  ^c^ Model 2: Delayed recall = CpG methylation + age + sex + education.  ^d^ “ε4”: *APOE* ε4 carrier status.  **P*<0.05, ***P*<0.01 | | | | | | | | | | | | | | | | | | | |

**Table S3.** Sensitivity analysis for the association between methylation and delayed recall in *APOE* ε2 non-carriers (N=183)^a^
