# Supplementary material for: DNA methylation in the APOE genomic region is associated with cognitive function in African Americans
Source: BMC Med Genomics. 2018 May 8;11:43. doi: 10.1186/s12920-018-0363-9 (PMC5941603; doi:10.1186/s12920-018-0363-9)
Supplement: Supplementary file 4 — Figure S1. Correlation of blood and brain methylation for the eight CpG sites significantly associated with delayed recall in GENOA: Results from the Blood Brain DNA Methylation Comparison Tool. Boxplots of methylation level by tissue type, and scatterplots demonstrating the relationship between methylation in whole blood and four brain regions (prefrontal cortex (PFC), entorhinal cortex (EC), superior temporal gyrus (STG), and cerebellum (CER)) in N = 71–75 matched samples from individuals archived in the MRC London Neurodegenerative Disease Brain Bank. Samples from both neuropathologically unaffected controls and individuals with variable levels of neuropathology were included. Plots were generated from the Blood Brain DNA Methylation Comparison Tool (http://epigenetics.essex.ac.uk/bloodbrain/). Only CpG sites that showed a significant association with delayed recall (FDR q < 0.1) in the GENOA sample were investigated. (PDF 595 kb) [file 12920_2018_363_MOESM4_ESM.pdf]

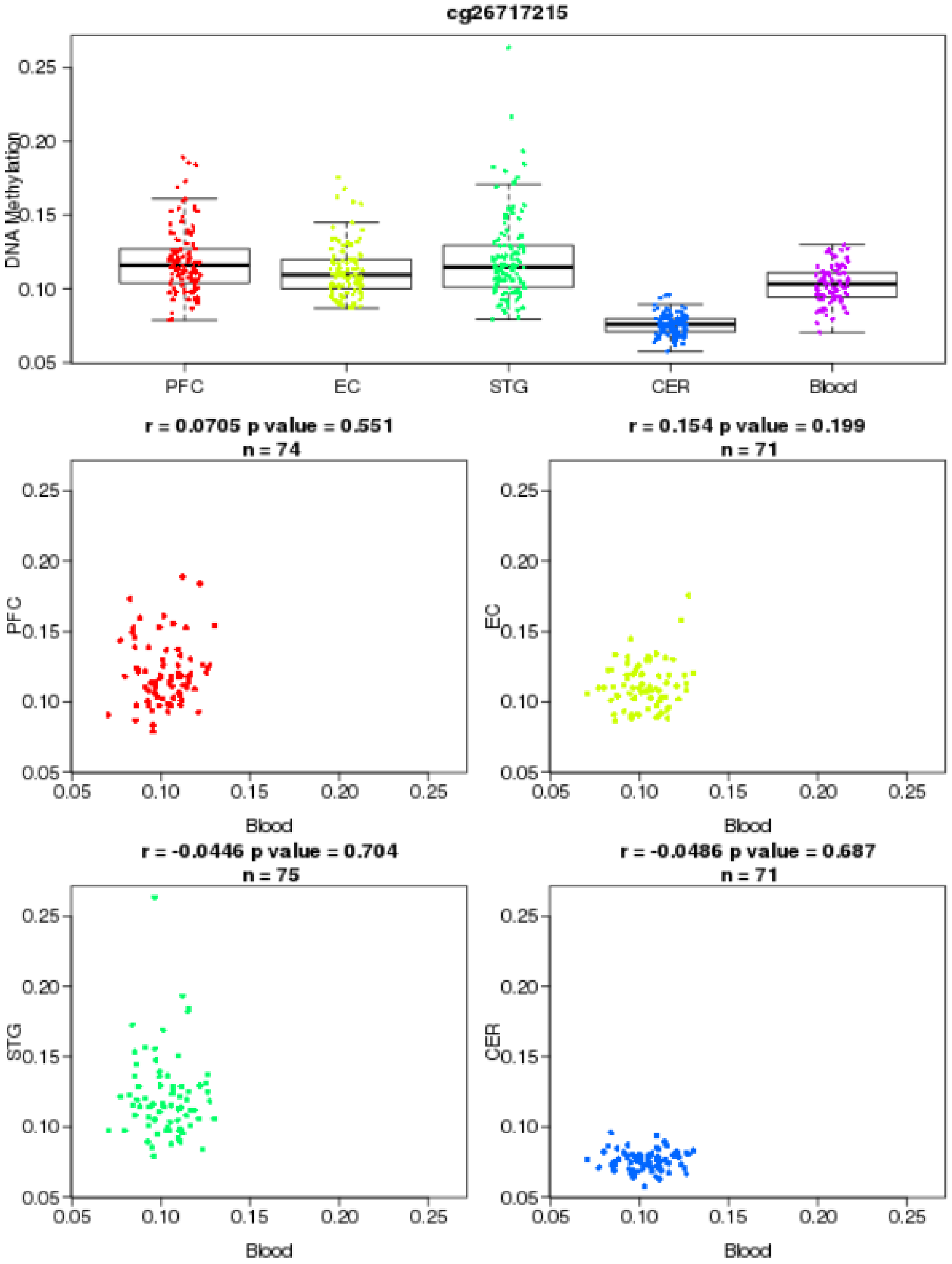

cg26717215
DNA Methylation
0.25
0.20
0.15
0.10
0.05
PFC
EC
STG
CER
Blood
r = 0.0705 p value = 0.551
n = 74
r = 0.154 p value = 0.199
n = 71
r = -0.0446 p value = 0.704
n = 75
r = -0.0486 p value = 0.687
n = 71
PFC
EC
STG
CER
Blood

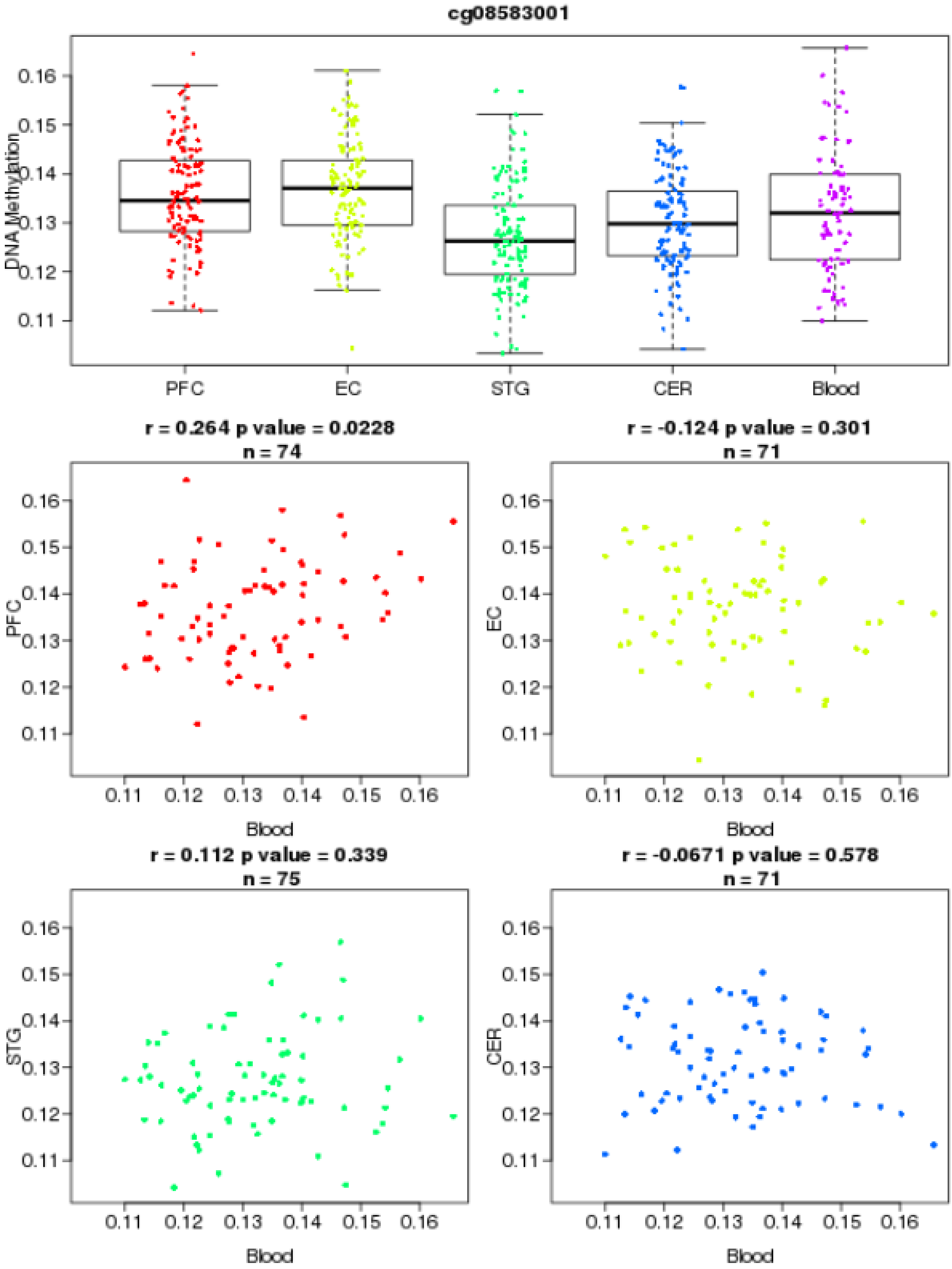
cg08583001
DNA Methylation
PFC
EC
STG
CER
Blood
r = 0.264 p value = 0.0228
n = 74
r = -0.124 p value = 0.301
n = 71
r = 0.112 p value = 0.339
n = 75
r = -0.0671 p value = 0.578
n = 71

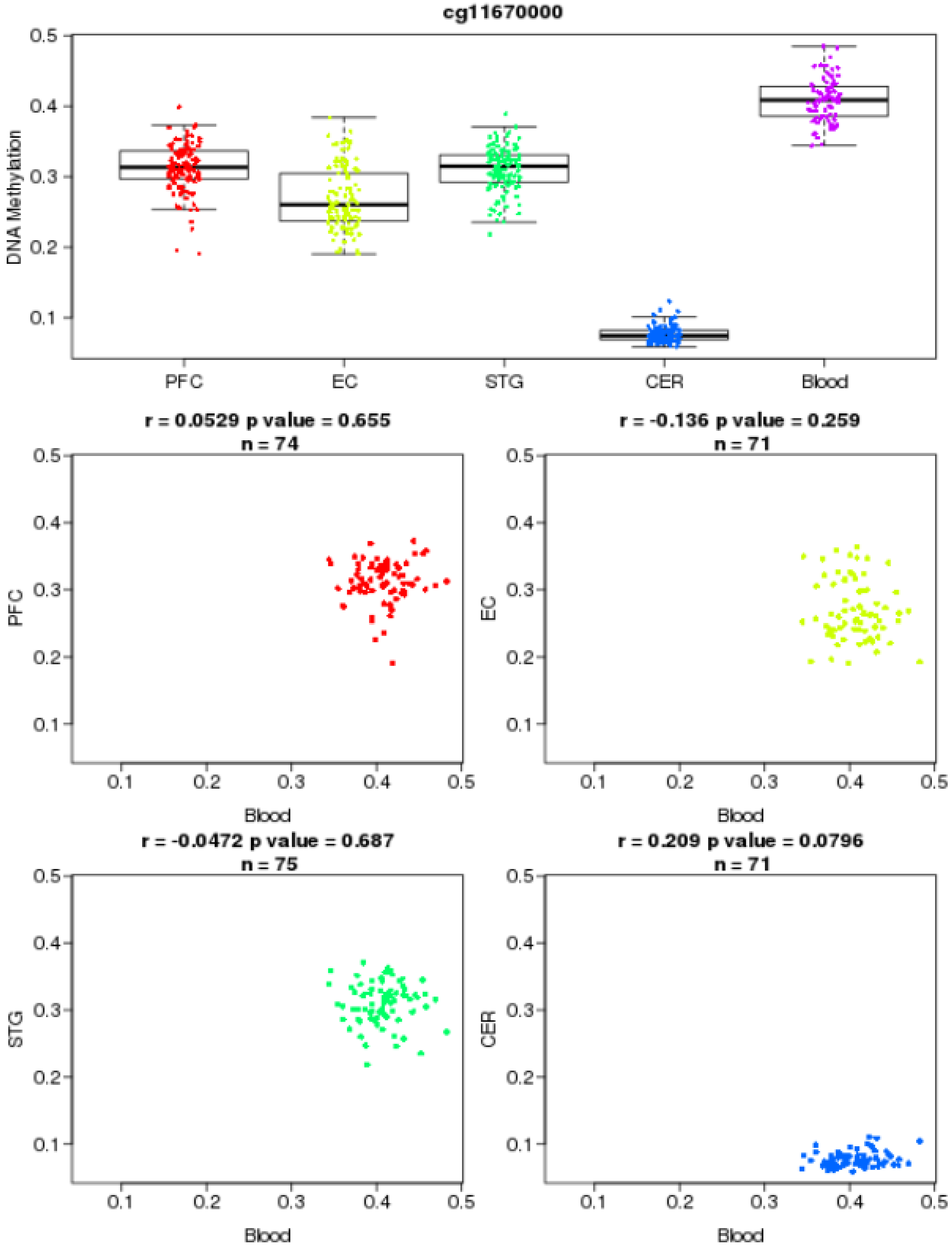

cg11670000
DNA Methylation
PFC
EC
STG
CER
Blood
r = 0.0529 p value = 0.655
n = 74
r = -0.136 p value = 0.259
n = 71
r = -0.0472 p value = 0.687
n = 75
r = 0.209 p value = 0.0796
n = 71

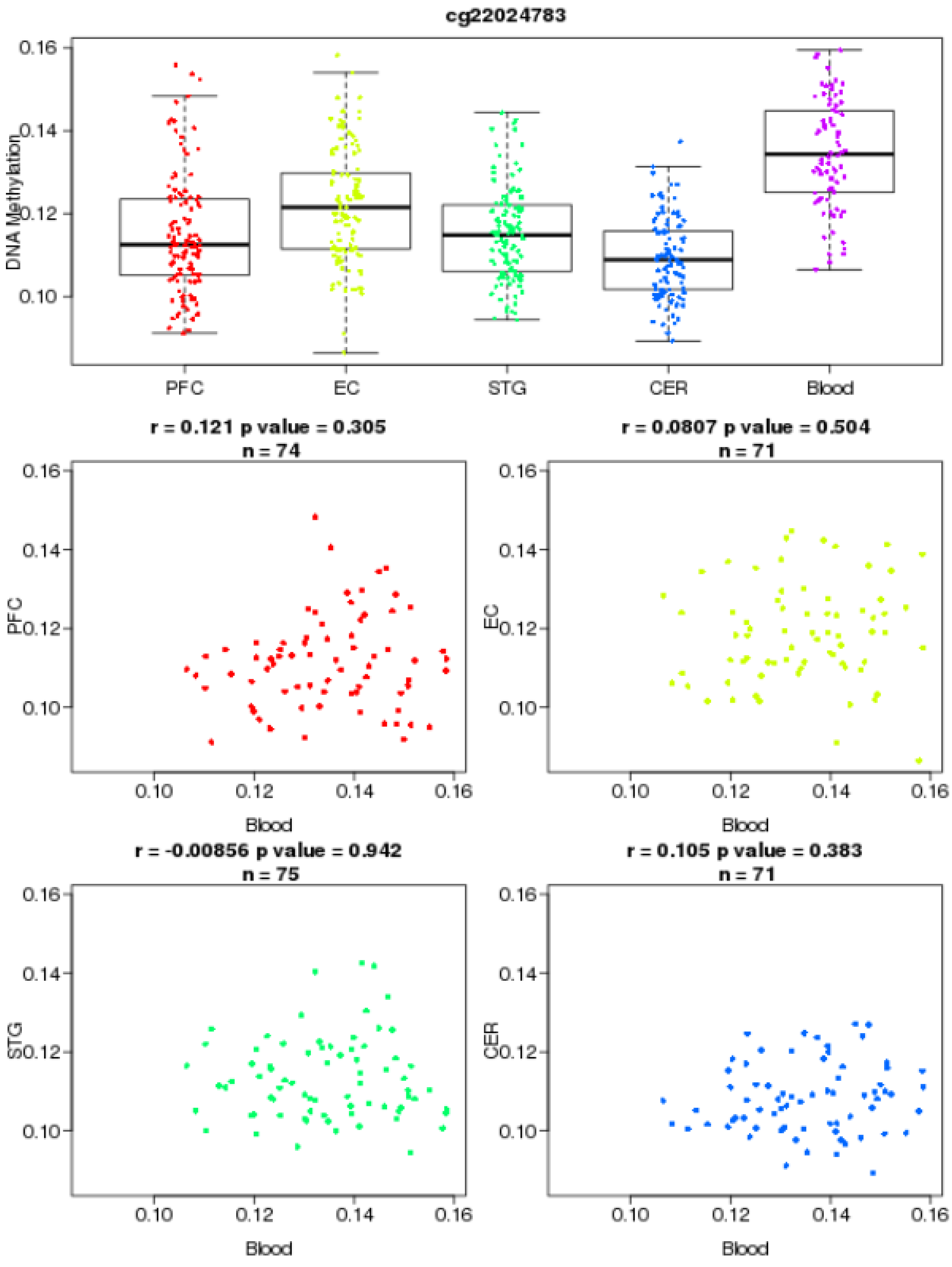
cg22024783
DNA Methylation
0.16
0.14
0.12
0.10
PFC
EC
STG
CER
Blood
r = 0.121 p value = 0.305
n = 74
PFC
Blood
r = 0.0807 p value = 0.504
n = 71
EC
Blood
r = -0.00856 p value = 0.942
n = 75
STG
Blood
r = 0.105 p value = 0.383
n = 71
CER
Blood

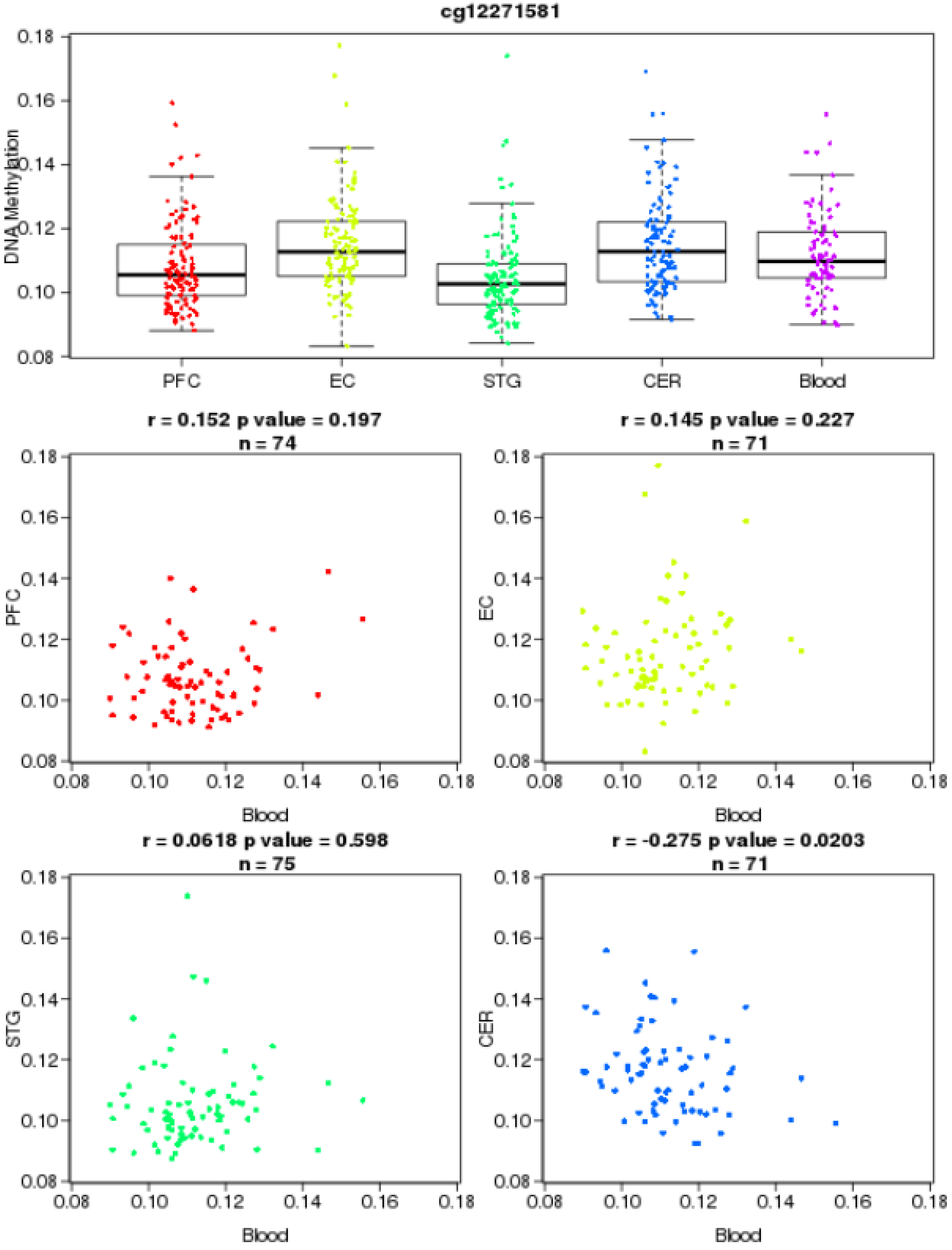
cg12271581
DNA Methylation
0.18
0.16
0.14
0.12
0.10
0.08
PFC
EC
STG
CER
Blood
r = 0.152 p value = 0.197
n = 74
PFC
Blood
r = 0.145 p value = 0.227
n = 71
EC
Blood
r = 0.0618 p value = 0.598
n = 75
STG
Blood
r = -0.275 p value = 0.0203
n = 71
CER
Blood

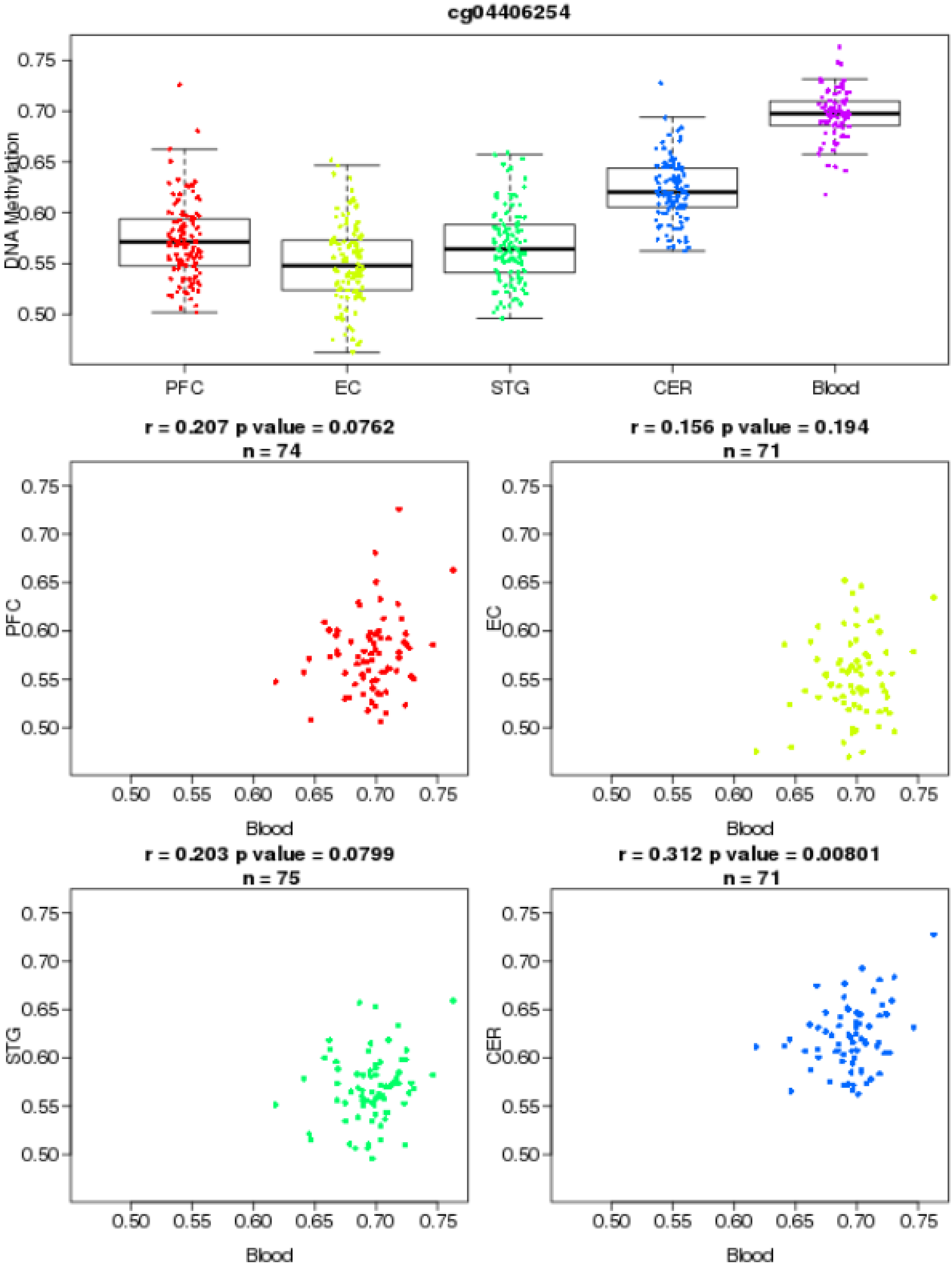
cg04406254
DNA Methylation
PFC
EC
STG
CER
Blood
r = 0.207 p value = 0.0762
n = 74
r = 0.156 p value = 0.194
n = 71
r = 0.203 p value = 0.0799
n = 75
r = 0.312 p value = 0.00801
n = 71

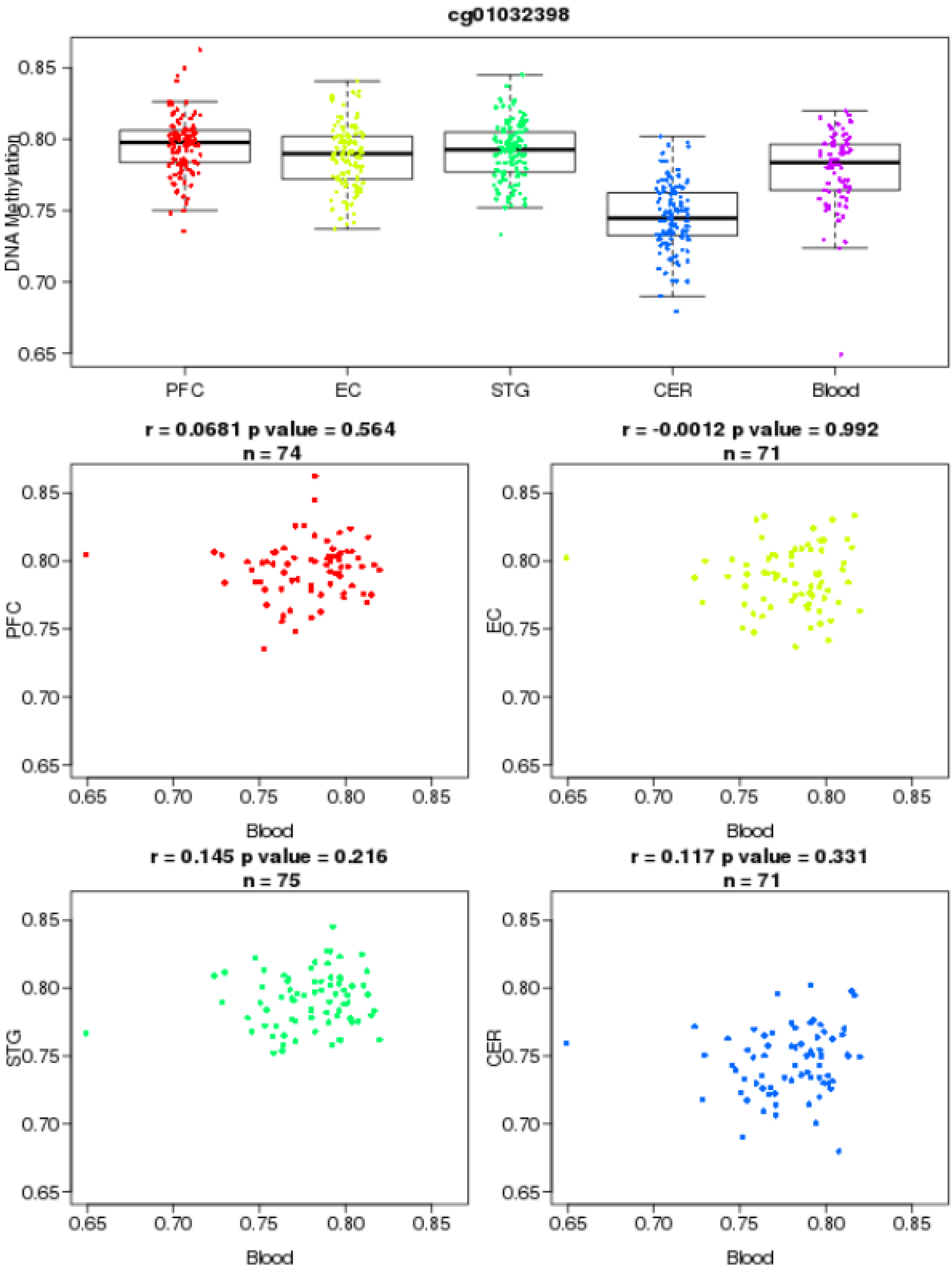
cg01032398
DNA Methylation
0.65
0.70
0.75
0.80
0.85
PFC
EC
STG
CER
Blood
r = 0.0681 p value = 0.564
n = 74
r = -0.0012 p value = 0.992
n = 71
r = 0.145 p value = 0.216
n = 75
r = 0.117 p value = 0.331
n = 71
Blood

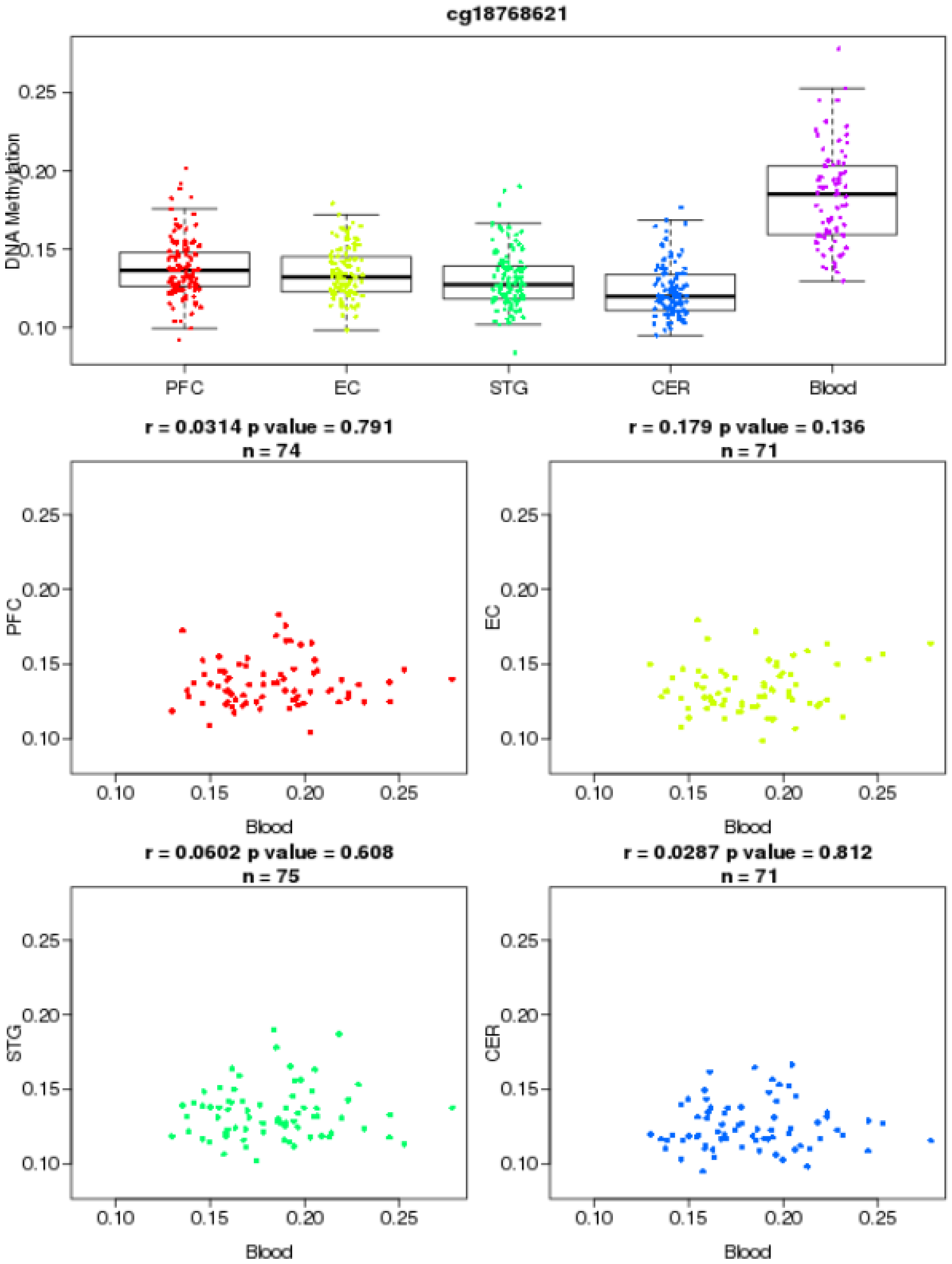
cg18768621
DNA Methylation
0.25
0.20
0.15
0.10
PFC
EC
STG
CER
Blood
r = 0.0314 p value = 0.791
n = 74
r = 0.179 p value = 0.136
n = 71
r = 0.0602 p value = 0.608
n = 75
r = 0.0287 p value = 0.812
n = 71
Blood
