# Supplementary material for: DNA methylation in the APOE genomic region is associated with cognitive function in African Americans
Source: BMC Med Genomics. 2018 May 8;11:43. doi: 10.1186/s12920-018-0363-9 (PMC5941603; doi:10.1186/s12920-018-0363-9)
Supplement: Supplementary file 5 — Figure S2. Correlation of blood and brain methylation for the eight CpG sites significantly associated with delayed recall in GENOA: Results from Blood-Brain Epigenetic Concordance (BECon). Comethylation plots showing the methylation levels in blood and three cortical regions (Broadmann area 10 (BA10), prefrontal cortex; Broadmann area 7 (BA7), parietal cortex; and Broadmann area 20 (BA20) temporal cortex) in 16 individuals ranging from 15 to 87 years of age from the Douglas-Bell Canada Brain Bank (panel 1), as well as a summary of blood-brain correlations (Spearman correlation value), variability of the CpGs (range of beta values between 10th and 90th percentile in the sample), and the effect of cell composition (change in beta value before and after adjustment for cell composition) (panel 2). Plots were generated from the Blood-Brain Epigenetic Concordance database (https://redgar598.shinyapps.io/BECon/). Only CpG sites that showed a significant association with delayed recall (FDR q < 0.1) in the GENOA sample were investigated. (PDF 21 kb) [file 12920_2018_363_MOESM5_ESM.pdf]

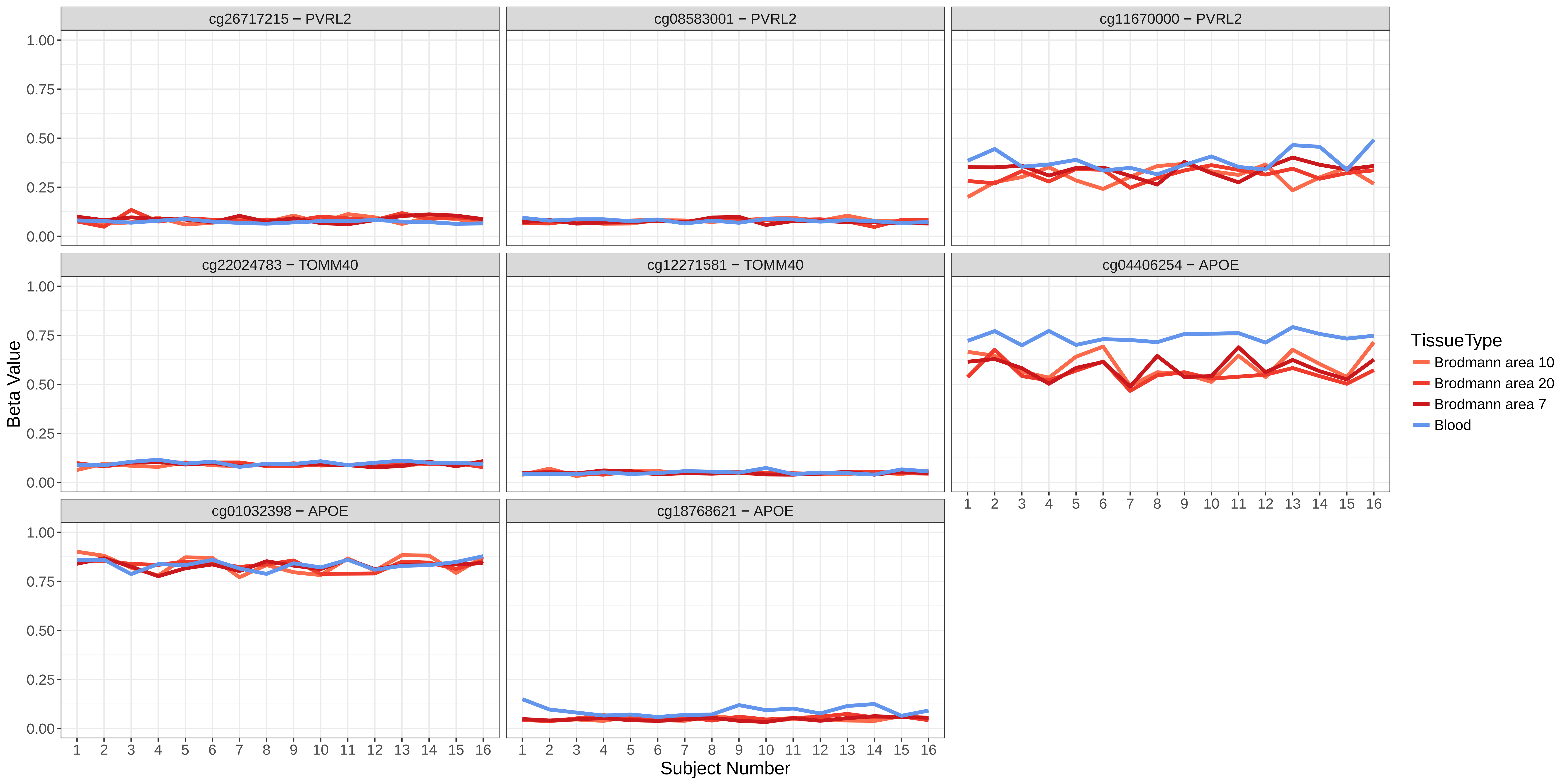
cg26717215 − PVRL2
cg08583001 − PVRL2
cg11670000 − PVRL2
cg22024783 − TOMM40
cg12271581 − TOMM40
cg04406254 − APOE
cg01032398 − APOE
cg18768621 − APOE
Beta Value
1.00
0.75
0.50
0.25
0.00
1 2 3 4 5 6 7 8 9 10 11 12 13 14 15 16
Subject Number
TissueType
Brodmann area 10
Brodmann area 20
Brodmann area 7
Blood

	Chr	Coor	Gene(s)	Gene Region(s)	Variability BA10	Variability BA20	Variability BA7	Variability Blood	Correlation BA10	Correlation BA20	Correlation BA7	Cell Composition Blood	Cell Composition Brain
cg26717215	19	45347921	PVRL2	promoter	0.04	0.04	0.04	0.02	−0.23	0.13	−0.32	0	0.01
cg08583001	19	45348226	PVRL2	promoter	0.03	0.02	0.02	0.02	0.07	0.15	−0.29	0	0
cg11670000	19	45352950	PVRL2	intragenic	0.12	0.07	0.08	0.12	−0.09	−0.56	0.45	0.04	0.03
cg22024783	19	45393916	TOMM40	promoter	0.02	0.02	0.02	0.02	−0.2	0.24	0.3	0.01	0
cg12271581	19	45394330	TOMM40	promoter	0.02	0.02	0.02	0.02	0.32	−0.34	0.24	0.01	0
cg04406254	19	45407945	APOE	promoter	0.16	0.09	0.12	0.07	0.24	0.11	0.03	0.02	0.03
cg01032398	19	45408121	APOE	promoter	0.1	0.05	0.05	0.06	−0.08	0.4	0.21	0.02	0.01
cg18768621	19	45409440	APOE	intragenic	0.02	0.02	0.02	0.06	0.22	−0.03	0.28	0.02	0

Correlation or Cell Composition Percentile or Variability Status

- 90% (Positive)
- 75−90% (Positive)
- 50−75% (Positive)
- <50% (Positive)
- <50% (Negative)
- 50−75% (Negative)
- 75−90% (Negative)
- 90% (Negative)
- 90% (Blood Cell Comp.)
- 75−90% (Blood Cell Comp.)
- 50−75% (Blood Cell Comp.)
- <50% (Blood Cell Comp.)
- 90% (Brain Cell Comp.)
- 75−90% (Brain Cell Comp.)
- 50−75% (Brain Cell Comp.)
- <50% (Brain Cell Comp.)
- Not Variable
- Variable
- Genomic Info
